# The politics of national SDG indicator systems: A comparison of four European countries

**DOI:** 10.1007/s13280-022-01809-w

**Published:** 2023-02-11

**Authors:** Robert Lepenies, Leonie Büttner, Ilona Bärlund, Kurt Jax, Jari Lyytimäki, Anders Branth Pedersen, Helle Ørsted Nielsen, Claire Mosoni, Raoul Mille, Gerard Payen, Didier Richard

**Affiliations:** 1 Environmental Politics Department, Helmholtz Centre for Environmental Research – UFZ, Permoserstr. 15, 04318 Leipzig, Germany; 2 Karlshochschule International University, Karlstraße 36-38, 76133 Karlsruhe, Germany; 3 Department Aquatic Ecosystem Analysis and Management, Helmholtz Centre for Environmental Research – UFZ, Brückstr. 3a, 39114 Magdeburg, Germany; 4 Department Conservation Biology and Social-Ecological Systems, Helmholtz Centre for Environmental Research – UFZ, Permoserstr. 15, 04318 Leipzig, Germany; 5 Environmental Policy Centre, Finnish Environment Institute, Helsinki, Finland; 6 Department of Environmental Science, Aarhus University, Frederiksborgvej 399, 4000 Roskilde, Denmark; 7 Climate Change Programme, Finnish Environment Institute, Helsinki, Finland; 8 National Academy of Technology of France, Paris, France; 9 l’Institut National de Recherche en Agriculture, Alimentation et Environnement (INRAE), Paris, France

**Keywords:** Environmental policy, Indicators, Indicator systems, Politics of monitoring, SDG indicators, Sustainability

## Abstract

The sustainable development goals (SDGs) constitute an ambitious comprehensive global framework including monitoring mechanisms and indicators to evaluate progress towards precise targets of sustainable development. Most European countries have adapted their national sustainability indicator systems to conform to the UN Agenda 2030 for sustainable development, introducing new indicators and monitoring frameworks and governance processes in which these are embedded. What do we know about the political processes and struggles of implementing this important global framework? How does the politics of indicators differ in national contexts? We propose a classification of national indicator systems along dimensions of indicator selection, appraisal landscape, participatory nature, and political communication. We empirically explore these dimensions for four European national sustainability indicator systems through a comparative analysis based on national policy documents, indicator databases, and web portals as well as inputs from workshops and expert interviews. Given the considerable variation with respect to the trajectory of national sustainability indicator systems, we posit that these differences correspond to different national interpretations of sustainability.

**Supplementary Information:**

The online version contains supplementary material available at 10.1007/s13280-022-01809-w.

## Introduction

Indicator systems have been central to global sustainability policies in the last decade, but it is only with the introduction of the SDGs that we see a global effort to streamline an indicator system across multiple levels of governance with the aim of connecting indicators to decision-making. Since 2015, the process of implementing a global monitoring framework toward the SDGs is an endeavor that has demanded considerable political and financial resources from national statistical agencies and international organizations. The underlying assumption of the process is that sustainability indicators are “key to decision-making” to bring about nothing less than “the transformation of our world” through a process of “follow-up and review” (United Nations 2015) of having achieved the goals and targets of the 2030 Agenda. They are a “management tool” and a “report card” which “help ensure the accountability of all stakeholders” (SDSN 2015, p. 2). Formal responsibility for measuring progress toward the SDGs—which requires high quality, accessible, and timely data collection—lies with UN Member States. From the start of the process it was expected that the global indicators “will be complemented by indicators at the regional and national levels which will be developed by Member States” (United Nations 2015).

Has this happened in Europe? There are a lack of comparison of national pathways to monitoring progress toward achieving Agenda 2030 beyond the technical level in Europe. There is little investigation of the question of whether European sustainability policies follow a similar understanding of what constitutes sustainable development and what the success conditions of indicator systems are. While statistical debates have evolved around questions of quality of data, data limitations or the scale of reporting coverage and methodological consensus, comparatively less has been written on the legitimacy of indicators, their political relevance, and stakeholder engagement (Pintér 2013). For this article, we dissect different political dimensions of national indicator systems. We argue that different political configurations surrounding indicator systems amount to nationally distinct ways in which sustainability policy is monitored and evaluated: this includes indicator selection, management, use in policies, and (mis)use in political debate as well as their appraisal. To do so, we start (Section “Conceptual framework, materials, and methods”) by introducing our framework and methodology. In Section “Results” we elaborate in detail case studies from four European countries, which are then compared and discussed in Section “Discussion: lessons learned from case studies”, before we end with some general conclusions (Section “Conclusion”).

## Conceptual framework, materials, and methods

### Political dimensions of national indicator systems

There has been increasing attention to understanding the political role that quantification and indicators play for the SDGs at the international level. Recent work especially by Rottenburg et al. (2015), Fukuda-Parr and McNeill (2019), and Bandola-Gill et al. (2022) has helped to understand the negotiation of power and knowledge surrounding the global SDG indicator framework. Work on the politics of SDG indicators has shown that indicator selection and governance is a highly political matter—contrary to its being described as technical and objective (Fukuda-Parr and McNeill 2019) which in turn allows for new spaces of engagement, participation, and contestation (Bandola-Gill et al. 2022). Indeed, Tichenor et al. (2022) describe these developments as the hyper-quantification along the dimensions of “new materialities, new interdependencies, and new governing ideas.” Despite these advances on understanding the politics of global indicator systems, less attention has been paid to how these indicators are being implemented in particularly national contexts (Mair et al. 2018).

Prior research has compared national sustainability strategies and respective assessment and monitoring approaches before the SDGs (Steurer and Martinuzzi 2007), with instructive conceptualizations of indicator systems for individual countries (Borgnäs 2016), while others have classified variance in sustainability indicator systems across Europe (Steurer and Hametner 2013). We are inspired by the above literature to construct a classification of the politics of national SDG monitoring which will help to, eventually, construct different descriptions of national indicator “cultures” in sustainability assessments.

We propose four dimensions that can serve as a heuristic against which indicator systems can be mapped (Table 1): (1) how they were selected, (2) who participates in their management, (3) how they are appraised and evaluated,^1^, and (4) who communicates them and how.^2^ With this, we follow Pintér et al. (2017) who urged to focus not only on “methods and instruments, but about its underlying subject—*what* is being measured, *why,* and by *whom*.”

**Table 1:** Characteristics of national indicator systems in their ideal types. Types denote a spectrum within which they can be positioned

Dimensions of national indicator systems		
**Selection** *Who selected indicators and was this selection understood as a political or technical endeavor*	**Technical** Indicators mostly selected by statisticians, understanding of indicators as neutral measurement tools	**Policy-driven** Selection of indicators resulting from either political deliberation or executive intervention
**Participation** *To what extent are stakeholders involved in ongoing sustainability strategy*	**Non-inclusive process** No or ad hoc involvement and participation, top-down processes	**Inclusive & citizen-based approach** Bottom-up participation as an integral part of indicator policy
**Appraisal landscape** *How are indicators reviewed, by whom and is this done formally or informally*	**Low Diversity of scrutiny** Little formal or informal appraisal, low level of reflection about indicators	**High diversity of scrutiny** Involvement of many formal and informal appraisals, peer review
**Communication** *Are indicators used as a tool of political communication by administration or other political actors*	**Low visibility** Indicators as a measurement tool, no policy actions	**High visibility** Indicators as a politically salient, highly debated, and contentious

**Table 2:** Evaluative summary of the national SDG indicator initiatives—case-specific features

	Finland	Germany	France	Denmark
*Selection process of indicators*	*Semi-open, data-driven process, yet involving a variety of stakeholders*	*Technical, non-inclusive, and data-driven approach*	*Goal oriented, explicitly political, inclusive and bottom-up approach*	*Policy-driven, non-inclusive in the beginning*
*Participation in indicator management*	*Innovative participatory elements (citizen panel)*	*Participation mostly from scientific community*	*Mostly ad hoc participation, statistical approach*	*Technical, data-driven approach, relatively non-participatory*
*Appraisal landscape*	*Highly sophisticated, mostly formal appraisals*	*Highly sophisticated with formal and informal appraisals yet commanding low awareness of political actors*	*Mostly formal appraisals*	*Variety of formal and informal appraisals commanding high awareness of political actors*
*Communication on indicators*	*Low visibility except for participatory experiments, some official effort to communicate*	*Low visibility, low effort to communicate*	*Low visibility except for wealth-related indicators, low effort to communicate*	*Low visibility, relatively low effort to communicate*

### Comparative analysis

In our study, we applied a comparative analysis to four national-level European sustainability indicator systems as they respond to Agenda 2030. The analysis is based on a mixed materials approach suitable for analyzing complex topics, such as national variations in sustainability indicator governance. Insights based on different data sources allow identification of key aspects while maintaining sensitivity to differences in contexts of indicator use and governance through case-oriented comparative methods (Ragin 1987). Our choice of two large high-income West European (France and Germany) and two smaller high-income Northern European countries (Denmark and Finland) that are all part of the European Statistical System (ESS) is based on a case selection logic of “most similar cases” (Teune et al. 1970). Our international author group with country-specific expertise responded to the same set of questions about indicator systems for all case studies (see Appendix S2). After receiving the first set of answers, we discussed the answers (bilaterally and within country groups) and then compared answers across countries, including more questions and dropping off others. In terms of data sources, we primarily used publicly available documents, indicator databases, and web portals describing indicator development on the international and national level (Appendix S3). Further steps of validation are also described in Appendix S3.

## Results

### Sustainability policy and the indicator system in Denmark

The Coordination Model of Sustainable Development in Denmark. Note: Content in this graph is primarily based on Rigsrevisionen 2020a).^3^

**Fig. 1:**
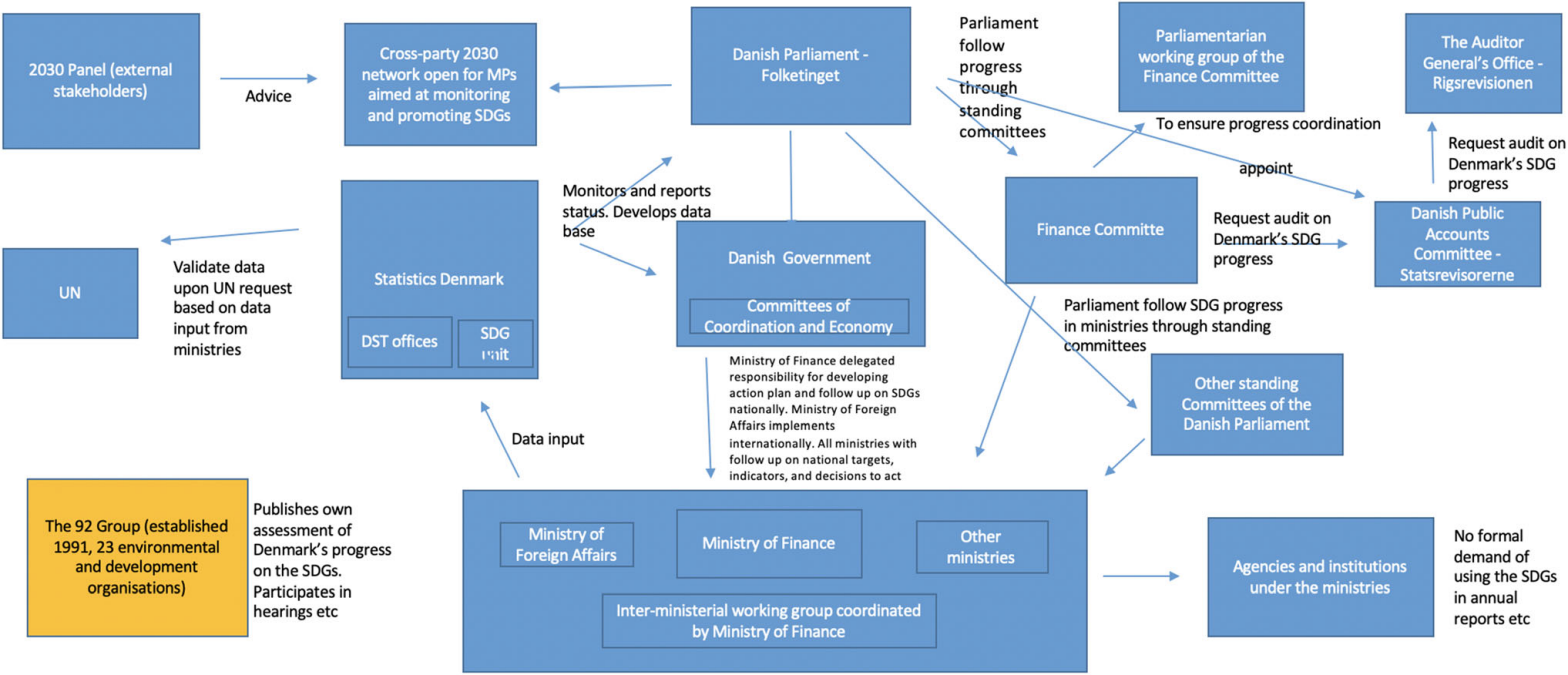
Management of SDG indicators in Denmark

Denmark translated the SDGs into a unique national indicator arrangement of 37 targets and 49 national indicators—a novel indicator system around which political actors negotiate different visions of sustainable development. Critiques or different interpretations of sustainable development were visible in the formal and informal appraisal mechanisms that we investigated. A first official voluntary national review (VNR) was presented in 2017 to the UN (Danish Government 2017b), while a progress report was published by the Ministry of Finance in 2018. The 58 pages report by the Ministry of Finance evaluates progress on each of the 37 national targets by drawing on some indicators. Two subsequent reports are planned for the period until 2030 (Ministry of Finance 2018). Progress is also reported at the website of Statistics Denmark.^4^ However, there was substantial critique of the Ministry of Finance 2018 report. In a closed hearing process, some powerful companies—e.g., Novo Nordisk A/S, Denmark’s largest company by market value—criticized the Liberal-Conservative Government for not conducting an open hearing process, for not considering the UN targets and indicators sufficiently and for ranking Denmark’s contribution to the SDGs alongside the government’s own political objectives. In particular, Novo Nordisk A/S criticized the government for politicizing the targets (Ritzau 2018). The same critique has been raised by other experts, who find that the Danish government has written a plan for something Denmark is already doing (Politiken 2018). The NGO ‘Globalt Fokus og 92-gruppen’—consisting of 23 environmental and development organizations—criticized the Government for ‘not taking the responsibility for achieving the SDGs seriously.’ The group finds, in general, that the nationally translated indicators in the 2018 action plan do not reflect the SDGs and their global indicator framework, as well as their transformative ambition. Regarding the progress report, it is a problem, according to the NGO, that no data are reported on 15 of the 47 indicators (Globalt Fokus and 92-gruppen 2018a; Ritzau 2018). Moreover, several organizations published their own shadow progress reports as a form of protest. A report which, according to the NGO, aims at evaluating progress instead of just evaluating state in comparison with other countries (92 Gruppen & Globalt Fokus 2018b). As a follow-up, the group has published smaller spotlight reports in 2019 and 2020 (92 Gruppen and Globalt Fokus 2019, 2020). Further, there exists a “2030 panel” of external stakeholders, which has an advisory role (see Fig. 1), and has, in collaboration with Statistics Denmark—based on workshops, debates, social media interaction and inputs from companies, organizations and citizens—in September 2020 suggested 197 Danish measure points as a supplement to the UN indicators (2030-Panelet 2020). Therefore, the national translation of the action plan is now gradually opened up to include more indicators.

The critique issued by companies and NGOs were echoed when the Danish Public Accounts Committee—Rigsrevisionen—was requested by the Finance Committee of the Danish Parliament to assist the committee with a professional and objective follow-up on progress made against the Danish achievement of the SDGs.

Rigsrevisionen (2020a, b) published a searing critique of Danish SDG implementation. While a complex governance framework has been built around the SDGs (see Fig. 1), policy implementation is weak or non-existent. Rigsrevisionen criticize that the Danish government mistakenly believed that “their general activities and policies already embrace the SDGs.” Indeed, ministries undertook a political and qualitative assessment and not one that was based on an analysis of achieving individual targets, with some indicators having only been measured in one year or not at all (Rigsrevisionen 2020a, b). This meant that unfortunately, “ministries have only in exceptional cases taken new initiatives or made particular plans to realize the SDGs” and that ministries and agencies have not been pushed to incorporate the SDGs in their work (2020a). Rigsrevisionen also finds that the model for assessing the implications of new legislation for SDG achievement has only been tested on four bills.

Finally, in general, Rigsrevisionen finds that Statistics Denmark’s reporting of SDG data to the UN has been satisfying, but “… the way in which data have been communicated is not well suited to inform the public of progress made in Denmark against the SDGs” (Rigsrevisionen 2020b, p. 5) and admonishes that there is a divergence between the data published by the UN and the data published by Statistics Denmark due to differences in the calculation methods. One of the reasons is that Statistics Denmark is using the very reliable Danish person register for calculating the size of the population, while the UN often is using population projections (Rigsrevisionen 2020a). The findings of Rigsrevisionen were validated through a 2019 interview with a public employee in the Danish Ministry of Environment and Food: At the time the government perceived the SDGs as a check list for the direction of the World and the single countries—however, measurable targets and indicators were missing. Thus, from the outset SDGs were seen as a recapitulation of already ongoing work in the Danish ministries (Interview Danish Ministry of Food and Environment 2019).

### Sustainability policy and the indicator system in Finland

**Fig. 2:**
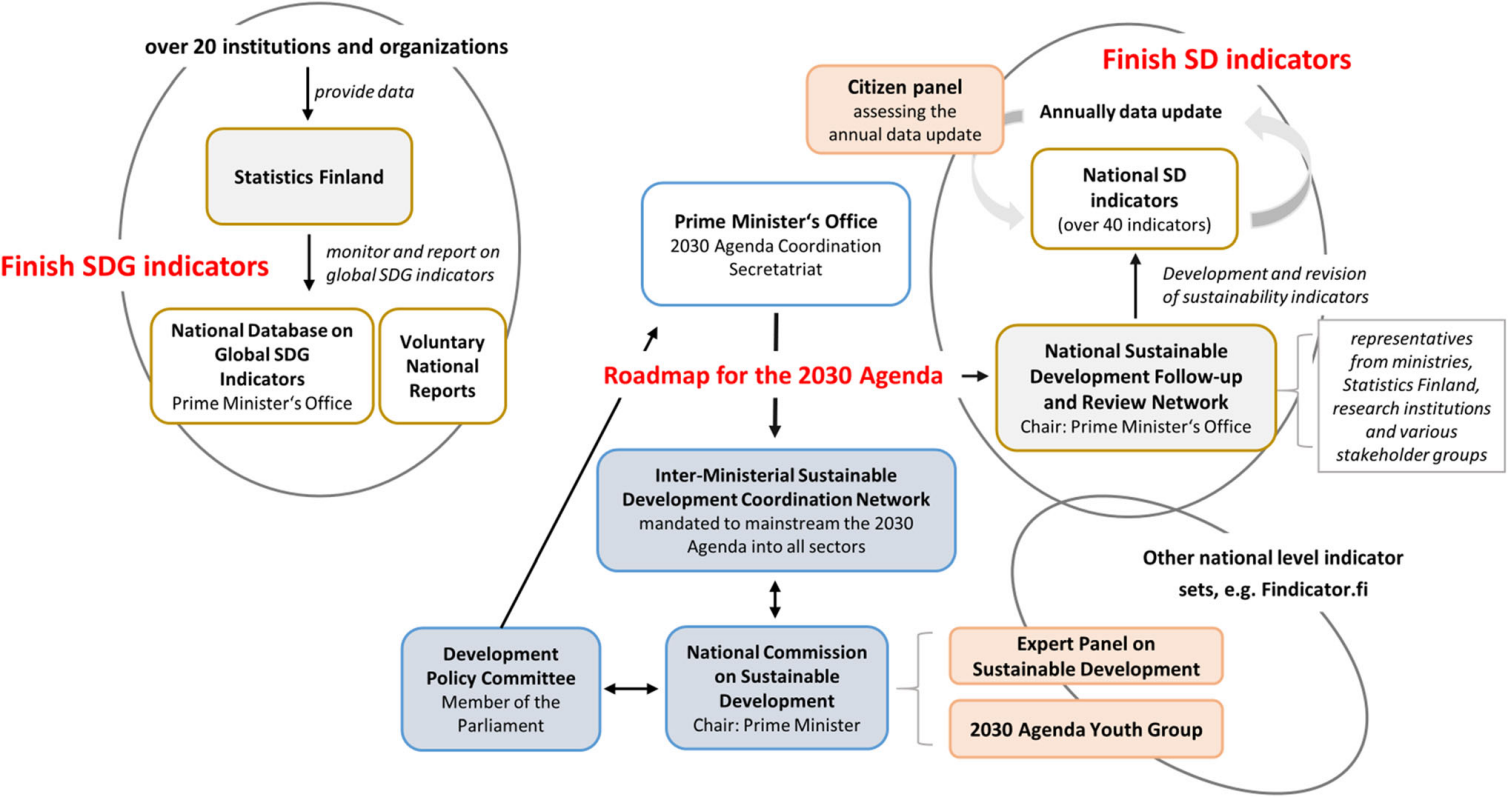
The coordination model of sustainable development policy in Finland

There are two official national-level sustainable development indicator sets: Finnish SDG indicators and national sustainable development indicator baskets (Fig. 2).^5^ The Finnish SDG indicator system is nearly congruent with the global SDG reporting system and does not construct national translations. In addition, the Prime Minister’s Office hosts a set of indicators tailored to follow the implementation of the Government Program and aimed to assist policy makers. An online service aimed for a broader audience called “Findicator” provides one hundred indicators of national-level societal progress. It has considerable overlaps with the SDGs. Furthermore, several other national-level sector-based indicator sets aim to serve more narrow knowledge needs related to issues, such as agriculture, education, or health.

After a committee report evaluating the national-level implications of the international Brundtland Report for Finland in 1989, several evaluations of sustainability policies have been conducted. These have ranged from independent studies (e.g., Rosenström 2009; Rouhinen 2014) to national evaluations mandated by Finnish National Commission for Sustainable Development (Patosaari 2003; Ramboll 2009; Berg et al. 2019). Since 2017, the Finnish Parliament has received the government’s yearly progress reports on Agenda 2030 and provided recommendations back to the government. In these progress reports, indicators played a considerable role as a basis of the assessment. The government presented an implementation plan for Agenda 2030 that identified key principles and focus areas for Finland (PMO 2017). Notably, the National Audit Office of Finland has performed two external independent national-level assessments (VTV 2010, 2019). In all of these reports, indicators were extensively utilized.

The Finnish national Expert Panel for Sustainable development, Kestävyyspaneeli, has been operating since 2013. It is an independent network of ten professor-level sustainability experts aiming to provide critical but constructive science-based advice. An Agenda 2030 Youth Group was established in 2017 by the National Commission for Sustainable Development in order to bring forward the views of the young. An online citizen jury consisting of volunteer citizens was organized (2019–2021) by the Prime Minister’s Office in order to collect the views of the citizens of the progress and priorities of sustainability. The citizens were invited to present their views regarding the development showed by national sustainability indicators. This panel represents a unique way of participatory assessment of SDG progress in the European context (P4R 2020) even though its effect has been limited (data limitations preventing the adoption of alternative indicators proposed by the panel). We also find that while the panel created a rich material, a lack of resources meant that the views of citizens could not be fully analyzed and utilized leading to some frustrations among participants. However, conclusions from the panel have been summarized and reported on by the Prime Minister’s Office. Because of resource cuts, citizen panel was not organized in 2022. In terms of non-formal policy appraisal, over the years, NGOs have published some critical thematic assessments related to SDGs (e.g., FINGO 2016).

Finland presented its first Voluntary National Review (VNR) to UN HLPF in 2016. The second VNR in 2020 included—innovatively—independent one-page reviews of each SDG by NGOs representing views of civil society (VNR 2020). The VNR preparation process included also an informal external peer review conducted by national experts from Switzerland and Mozambique in April 2020 (organized online due to COVID-19 restrictions on traveling).

Despite the intensive use of indicators to support sustainable development policies, the use in other policy domains remains limited (Rosenström 2009; Rinne et al. 2013; Berg et al. 2019). The direct policy use of indicators has been limited to the meetings of the National Commission for Sustainable Development and other activities directly related to implementation of sustainable development policies, such as national reporting to the UN. Persuasive use of indicators in communication processes such as speeches by policy makers has been more common—indicators have also served as conceptual aids for learning or enlightenment (Rinne et al. 2013). In particular, interviewed civil servants emphasize the role of sustainability indicators to generate overviews capable to break siloed approaches. The use of UN-based SDG indicators has so far been limited. For example, the government-mandated national assessment used national sustainable development indicators and international indicators as presented in the SDG Index and Dashboard to build an overall picture of the development in Finland (Berg et al. 2019). UN-based SDG indicators were not used in this assessment because of lack of comprehensive and readily available indicators. The national indicators have been used to support the monitoring, reporting, and assessment through annual high-level stakeholder events probing the state and future of sustainable development in Finland, coupled with the EU Sustainable development week (PMO, 2022).

Currently, somewhat confusingly, the indicator work in Finland is characterized by co-existence of two sets of indicators with partially different purposes and target audiences but a shared mission to holistically describe the national situation. The preparation and communication processes of these two indicator sets have remained independent from each other. Nevertheless, the shared sustainability vocabular is likely to create a risk of confusion since most target audiences lack the knowledge about the different contents and origin of the sets in, respectively, the national and the UN-based sustainable development strategies and target settings. However, the risk of confusion is decreased because of low media, public, and political visibility of both indicator sets. The poor general-level appeal of sustainability indicator sets is a problem that has been repeatedly noted (Rosenström 2009; Rinne et al. 2013; Lyytimäki 2019b). It is an open question whether the national sustainable development indicators gain more momentum after the release of a new national sustainability roadmap accepted on February 7, 2022 by the National Commission for Sustainable Development.

### Sustainability policy and the indicator system in Germany

**Fig. 3:**
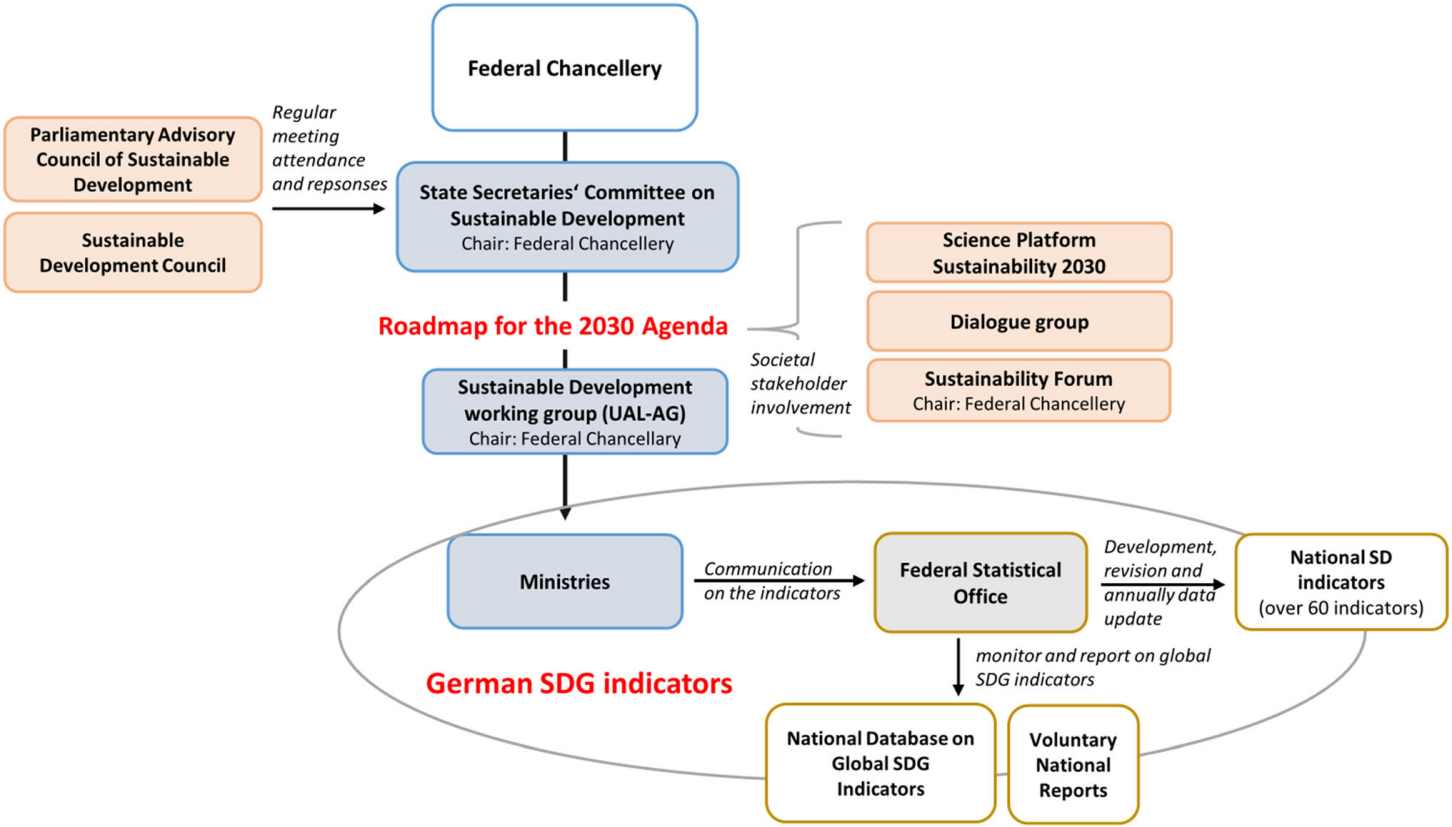
The coordination model of sustainable development in Germany

Germany developed its first sustainability strategy quite late, in 2002. However, it aligned and completely overhauled its strategy with the SDGs in 2017 (Fig. 3).^6^ A new sustainability strategy, including sustainability indicators, was adopted by the German government in early 2017. The sustainability governance structure with the relevant institutions—in which the implementation of the Agenda 2030 and the monitoring of SDG indicators are embedded—was already in place in 2015. Still, formal institutional structures have only been strengthened by the adoption of the Agenda 2030 and official commitment to the SDGs through a complete overhaul of the national sustainability strategy.

In Germany, the national sustainability strategy has been revised every four years since 2002, and in the meantime the sustainability policy and strategy has been evaluated through various actors, particularly the independent German Council for Sustainable Development (Rat für nachhaltige Entwicklung, RNE;) as well as the Parliamentary Advisory Council for sustainable development (PBnE) in the form of official appraisals. At this point one should mention in particular the international peer review, which has been implemented three times so far (2009, 2013 and 2017). The peer review is a formal appraisal mechanism commissioned by the German Council for Sustainable Development. Such an international peer review is only used by very few countries (Finland is among them). A consultation process organized by the German Science Platform (“wpn2030,” Wissenschaftsplattform) took place with the support of the Federal Government in the preliminary stages of rewriting or updating the German Sustainability Strategy, aiming to include the scientific community in the process. These networks are complemented by networks of sustainability experts in Germany that are situated between formal and informal appraisal mechanisms (e.g., the wider SDSN network) with individual experts at the science–policy interface being regularly consulted by the German chancellery.

There is only little parliamentary debate about the sustainability strategy in general and about the indicators in particular. An exception is the recent proposal by the Green Party that proposes wide ranging structural changes to German sustainability policy, using the strategy as an overarching policy framework, suggesting new indicators and asking for a mandatory legislative SDG impact assessment and a new SDG control council, drawing on many suggestions made also in PBnE (Green Party 2020).

According to the experts interviewed, public awareness and political relevance of sustainability indicators are increasing (Interview BMU 2020). Indeed, public debates are often framed around the sustainability indicators: See here The Parliamentary Advisory Council which demanded that the German government use sustainability indicators as a central instrument for policy-making and for determining future priorities on the basis of the (off-track) indicators (PBnE 2018 but see also in 2020). Apart from parliamentarians, it is also the German government itself which sees the indicators at the core of the SD management system (Federal Government 2016). The SDG indicator framework helps to implement and justify policy interventions (Interview BMU 2020). However, the extent to which the SDG indicator framework is taken seriously and used depends on which ministry is assigned responsibility for specific indicators. Indeed, it is particularly the Federal ministry of the Environment (BMU) and its associated agency (UBA) which uses sustainability indicators as strategic instruments in inter-ministerial negotiations. There is hope that that indicators indeed could hold other actors accountable (Interview BMU 2020).

At the same time, the new strategy makes a remarkable statement about the role of indicators in the new strategy: “Appraisals of the German Sustainability Strategy […] often focus on indicators. However, these are only a means to an end. What is ultimately important for the political debate, however, are the targets that determine the level of ambition for sustainable development [“Ambitionsniveau”], as well as the corresponding measures defined in the German National Sustainability Strategy (DNS). The number of sustainability indicators in the strategy is deliberately limited in order to provide an overview of the state of sustainable development with as little data as possible and thus to create a somehow manageable compass for sustainability policy.” (DNS 2021, our translation).

We obtained very similar results when surveying the opinion of indicator experts, who argued in interviews that there is currently too much focus on the monitoring and reporting system of SDGs in Germany according to the motto “We can only manage what we measure” (Interview with BMUB 2020). In the process, the implementation level is receding into the background (recognizable by the lack of financial support, the lack of a separate catalogue of measures, the lack of prioritization, the lack of human resources for implementation, etc.). In Germany, much of the technical discussion in statistical circles revolves around indicators and particularly about them being “off-track.” However, such discussions are nowhere paired with discussions about necessary policy interventions. This has also not changed in the update of the 2021 strategy.

### Sustainability policy and the indicator system in France

**Fig. 4:**
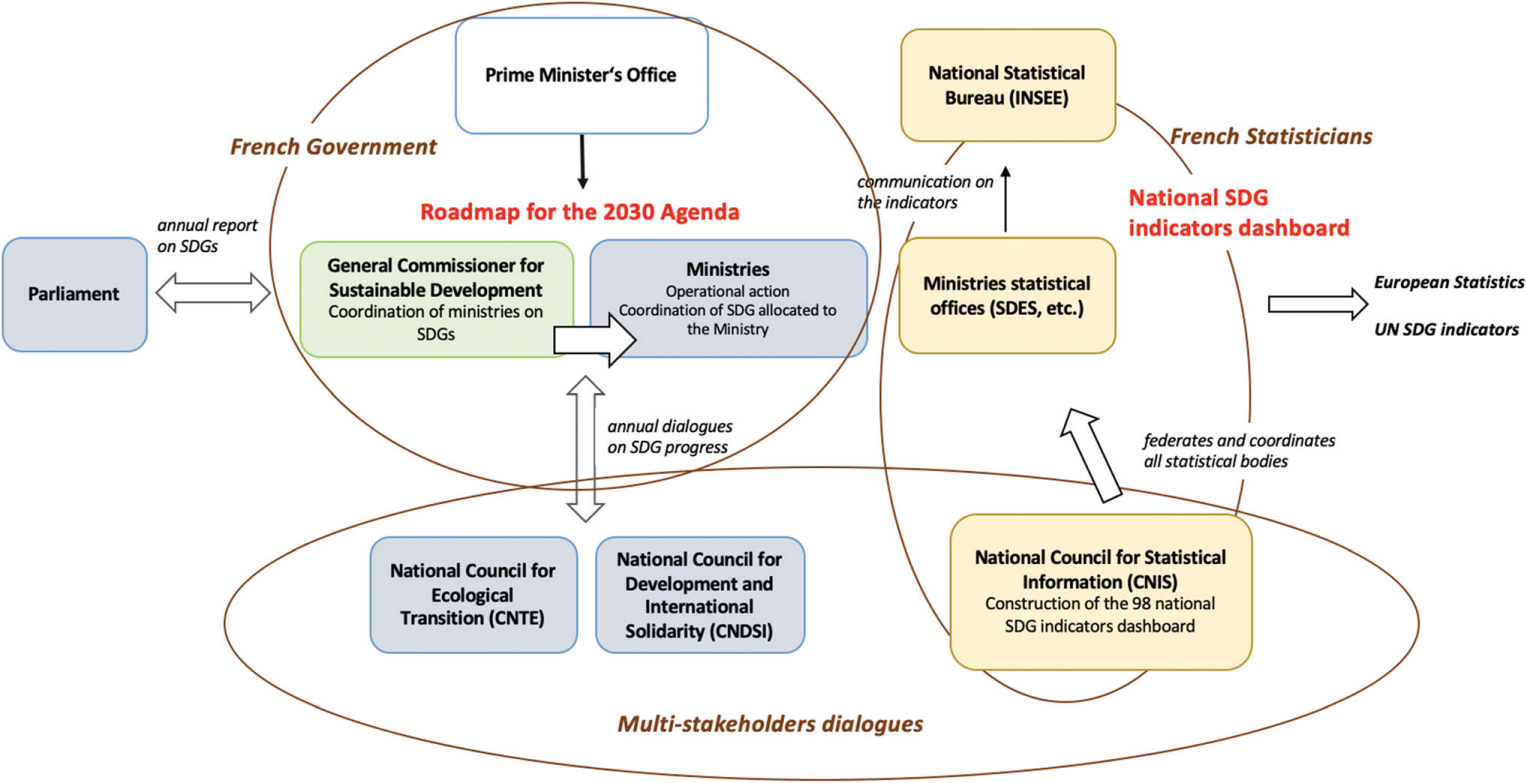
The coordination model of sustainable development in France

In France, the national preexisting sustainability strategy revised some months before the establishment of the Agenda 2030 and the SDG framework in 2015 (Fig. 4),^7^ was called the national strategy for an ecological transition to sustainable development 2015–2020—La stratégie nationale de transition écologique vers un développement durable (SNTEDD).^8^ It was adopted by the Council of Ministers in February 2015, about half a year before the adoption of the UN resolution on Agenda 2030 and the SDG framework by the United Nations (September 2015). Deliberations about a new French SDGs strategy started separately in 2017 (Aubert et al. 2017)—as the SNTEDD provided a roadmap only until 2020 and was mainly ecologically-oriented—this new strategy included the objectives of the SNTEDD as well as other (socio-economic) sustainability objectives, This new document was released in September 2019 and represents France’s new roadmap^9^ for the 2030 Agenda.

Sustainability indicators followed a similar evolution as the national sustainable development strategies. Indeed, there was first the establishment and development of national sustainability indicators linked to the SNTEDD as well as the national strategies that came before. The establishment of national SDG indicators came after the release of the global SDG indicators in 2017 and the French decision to draw a new roadmap implementing the SDGs (Interview Ministère de la Transition Ecologique or Ministry for the Ecological Transition 2020).

In fact, since the first national sustainable development strategy in 2003, national indicators had been continuously revised throughout the years, starting already in 2003 with 45 original national sustainability indicators implemented by the predecessor of the SDES *(Data and Statistical Studies Service of the French Ministry for the Ecological Transition)* in collaboration with INSEE *(National Institute of Statistics and Economic Studies)* (Bovar et al. 2008). In 2006, those indicators were updated to match the European strategies which defined 12 key SD indicators (Bovar et al. 2008). Finally, in 2015, the CNTE (*National Council for Ecological Transition*) set up a commission responsible for developing national indicators for ecological transition and the green economy in order to follow the SNTEDD. At the end of a concerted process, this commission defined 72 national SD indicators (Boulin and Vey 2017). To those then were added ten new wealth indicators which are specific to France and aimed to surpass GDP as a central indicator (see Appendix S4). They have a very important role as the law n° 2015–411 of April 13, 2015 requests the government to submit to each annual budgetary year a report concerning those ten wealth indicators (Aubert et al. 2017).

The official appraisal mechanism for evaluating progress toward the SDGs is coordinated by the General Commissioner for Sustainable Development (CGDD) housed in the Ministry for the Ecological Transition in connection with the Ministry of Foreign Affairs (Interview MTE Ministry for the Ecological Transition 2020). It reports annually to both, the CNTE and the National Council for Development and International Solidarity (CNDSI), two instances that monitor progress made in their respective thematic domains. This coordination by CGDD is needed because in France, once the strategy is in place, different ministries are responsible for the SDG implementation related to their thematic responsibility. Another monitoring measure is the yearly progress report on implementation made for the Parliament (Interview Ministry for the Ecological Transition 2020). In all of these documents, indicators play an important role: the monitoring of the national strategy is implemented through the 98 national SDG indicators—most of which are not new but selected on the basis of data availability and existing records. Those indicators are considered to be “at the heart of the roadmap monitoring system” (République Française 2019) This list of indicators is also intended to evolve in future, in particular by integrating indicators of absolute poverty. A reference website (https://www.agenda-2030.fr/) has been created for presenting the SDGs and the SDG targets, accessing to French values of national, European, and global SDG indicators, following the national SDG strategy and the achievement of the 2030 Agenda (Interview Ministry for the Ecological Transition 2020). With regards to civil society actors, SDG watch notes that there is “no structured and institutionalized participatory mechanism allowing regular NGO input to the French SDG implementation “with public consultations consisting of “ad hoc open meetings” with little input to the voluntary national review, indeed “no national civil society SDG coalition exists in France”—with Association 4D and WECF-France “currently working to initiate” such a coalition (SDG Watch 2021).

The role of SDG indicators is to support the monitoring of the SDGs implementation in France and, more widely, the evolution of all sustainable development related policies (Interview French Partnership for Water 2020PFE). Indeed, at the institutional level, SDG indicators are used for monitoring the strategy with an annual report to the French Parliament. Indicators are quite visible in the SDG discussion and since the implementation of the new roadmap, there is no longer any public debate on the subject of indicators or their role. However, it is now the implementation of the SDGs within this roadmap by all actors (local authorities but also companies) which is to be improved. This implementation within the local authorities has to be adapted to the new national SDG indicators frame (Interview Ministry for the Ecological Transition and French Partnership for Water 2020).

## Discussion

### Lessons learned from case studies

The main result of our comparative study is that there exist significant differences in national pathways toward monitoring (and not just implementing) Agenda 2030 in the four analyzed European countries. These may mirror national differences in conceptualizing sustainability. At the same time there is also a marked convergence between European countries: nearly all countries have reformulated their national sustainability strategies and have constructed or adapted national sustainability indicator systems as a response to Agenda 2030. The number of chosen national SDG indicators has been limited to under 100 in all four country case studies (from 47 in Denmark to 98 in France, with 66 in Germany^10^). When asked, public authorities argue that these national translations do not replace, but do indeed complement global SDG indicators (which are far more numerous and were designed to fit global SDG targets). For all countries, three levels of SDG indicator systems coexist: the global, the European, and the national systems. Yet on top of these, there are national sustainability strategies that more or less align with these indicators. The level of public debate about indicators of the respective sustainability or SDG strategies varies significantly in the different countries, though, and varies also between parliamentary debates, civil society interventions, and statements by policy makers. While official review processes have had a limited impact in all countries surveyed, we see significant impact from informal appraisal mechanisms that will shape different national SDG implementation going forward (Table 2).

SDG indicators were supposed to be “the backbone of monitoring progress toward the SDGs at the local, national, regional, and global levels” (SDSN 2015, p. 7). While SDG indicators form an indispensable part of all sustainability strategies in our cases (with the exception of Denmark), it is not clear that this objective has been met. We observe fragmentation of indicator systems, with similar limitations in all countries: data gaps, insufficient links to policy implementation, and low overall visibility. The risk is that national indicator systems might divert attention away from global monitoring (Denmark, Germany) or mean that UN and national statistical bureaus might have different calculation methods (Denmark). Yet, if countries use the UN SDG’s indicator framework, there is a risk of confusion due to different indicator systems among experts, policy makers, and the public (Finland).

We also observe a proliferation of indicators and indicator sub-systems across different governance levels in all cases since 2015: not only do different political actors attribute different meanings to the same (or similar) indicators—we find that even indicator experts are confused as regards overlapping, duplicate, or incomplete monitoring and evaluation systems within and across different countries. Future research should investigate whether this proliferation is also leading to a similar weakening of transformative ambition as has been criticized by Fukuda-Parr and McNeill (2019) for the case of the global indicator framework.

This is relevant for further analyses of the politics of indicators going forward: indicators are often thought of as complexity-managing or -reducing devices: with the proliferation of sustainability indicators at different levels of governance, we find that the reliance on SDG indicators could, paradoxically, contribute to rising complexity: be it due to parallel indicator sets (Finland), indicator sets emerging from different sustainability prioritizations (France), or indicators that are promoted by specific political parties in power (Denmark), or indicator processes that are relatively opaque (Germany). We need to revise the overly optimistic assumption that a single, global, conceptual framework leading to uniform indicator systems providing easily comparable information would be possible.

In general, national sustainability indicators seem to be relatively poorly visible in the eyes of the public. This relative public invisibility of indicators is somewhat surprising, given the continuous reminder of their importance by sustainability scientists and given repeated official publications showing that SDG indicators are severely “off-track” (EEA SO 2020). The same is true for policy makers. We find that in general in our case studies, sustainability policy is not framed in the language of the SDGs or through invoking national sustainability strategies, nor are national SDG indicators used to inform these debates. A similar picture emerges when comparing parliamentary debates: the SDGs are not a salient political issue in any of the countries investigated, and, with the exception of Finland, little political capital is currently invested in communicating national sustainability strategies. In general, there is a low political salience of the SDGs and indicators.

At the same time, sustainability indicators act as boundary objects between a growing number of actors. SDG indicators are no longer negotiated only between scientists and policy makers, but also between and within statistical agencies, supreme audit institutions, nongovernmental organizations and parliamentarians, and participatory initiatives at increasingly diverse boundaries. What indicators mean is changing and in flux. Ideally, this allows that the indicators “are both plastic enough to adapt to local needs and constraints of the several parties employing them, yet robust enough to maintain a common identity across sites” (Star and Griesemer 1989, p. 393). In the best case, this unexpectedly contributes to SDG 17 (“Build transformative partnerships”) by bringing together new partners and sectors that hold each other accountable. At worst, it fragments sustainability policy and dilutes the ambition of the 2030 Agenda.

An interesting finding across all cases is the powerful role of auditing institutions and shadow reports in shaping indicator discourse. We find that formal—and particularly informal—appraisal mechanisms shape national SDG monitoring systems. While all countries are invited to submit VNRs as one form of appraisal, governments have used this opportunity with different degrees of commitment. Finland, for example, invited a peer review process (in Germany, this was taken up by the academic community in particular). In Denmark, a lively debate took place following shadow reporting by the NGO Globalt Fokus and 92-gruppen. In Finland, innovative forms of peer review were only slightly held back due to lack of funding. In France, only very sparse appraisal mechanisms exist because SDGs are not yet explicitly mentioned in sectoral policies. A common and strong finding is the existence of a surprisingly incisive peer review by supreme audit institutions in all countries except France (Rigsrevisionen in Denmark, Bundesrechnungshof in Deutschland, Audit office in Finland).

At the same time, there are several governance innovations surrounding national indicator systems which show the potential for cross-national learning: national indicators could indeed be instruments of transformative change and can open up spaces which can be creatively filled by actors with transformative interests: see the example of “Schlüsselindikatoren” in Germany, the “Findicator” in Finland, the support of alternative wealth indicators in France, and the suggestion of new measure points in Denmark from the 2030 panel and Statistics Denmark. SDG indicators are highly malleable and are translated differently in different national contexts—in quite surprising ways—indeed opening up windows of opportunity for new voices to be heard in sustainable development policy.

Variance in the participatory nature of indicator processes is demonstrated by looking at the differences in national indicator selection. In some countries, this was a largely top-down political process (Denmark), while in others, it was largely left to statisticians, who agreed on them in consultation with the relevant ministries, without public discussion accompanying the selection process (Germany). In France and Finland, on the other hand, serious efforts were made to involve not only statisticians in the initial development of a new set of indicators and thus to create a political discussion about the indicators. To pick one innovative example: In Finland, participation in indicator processes is especially significant in the annual evaluation: When data for the national SD indicators are updated, it is underpinned with an interpretative text by experts from ministries and research and then evaluated by a Citizen Panel.

## Conclusion

Sustainability indicators are linked to integrated and embedded into diverse sustainability policy strategies at the national level in various ways and are negotiated and interpreted by a variety of political actors. Understanding this allows us to not pin false hopes on indicators alone. We find considerable variation in the political dimensions of national indicator systems as different European countries respond differently to the challenges of monitoring progress toward Agenda 2030. More research is needed to understand exactly at which point these differences turn into obstacles to implementing Agenda 2030—and where they open up spaces for deliberation and transformation. We find that currently, there is a proliferation of indicators across governance levels and scales (though this only sporadically reaches to local levels). Here, we explore related risks and opportunities. We observe how sustainability indicators in SDG strategies function as boundary objects, around which different actors negotiate understandings of sustainability. While it is too early to say to what extent indicators stimulate concrete policy action, we do see the SDGs altering sustainability strategies. We highlight instances where indicators have been embedded in new forms of participatory governance arrangements or where they have increased political accountability. We indicate where political actors have embraced them as instruments that help steer toward sustainability transformations. We discovered a remarkably varied and influential appraisal landscape for indicators: we find that informal appraisals (and new appraisers such as national audit institutions) have substantially impacted national sustainability strategies—a fact that countries with yet underdevelopment appraisal mechanisms should take note of National and global SDG indicators, while heralded as the key to SDG implementation have not yet galvanized the action necessary to transform our world toward sustainability.

## Supplementary Information

Below is the link to the electronic supplementary material.

Supplementary file1 (PDF 838 kb)

## Footnotes

1 See Appendix S1 for examples of formal, shadow and hybrid appraisal mechanisms.

2 We define the level of communication about indicators as a composite of different aspects: the public visibility of indicators, the way in which political actors use SDG indicators in arguments and the way in which public agencies rely on indicators in their day-to-day operations.

3 A full description of the sustainability policy and the indicator system in Denmark can be found in Appendix S4.

4 https://www.dst.dk/da/Statistik/Sdg.

5 A full description of the sustainability policy and the indicator system in Finland can be found in Appendix S4.

6 A full description of the sustainability policy and the indicator system in Germany can be found in Appendix S4.

7 A full description of the sustainability policy and the indicator system in France can be found in Appendix S4.

8 https://www.ecologique-solidaire.gouv.fr/strategie-nationale-transition-ecologique-vers-developpement-durable-2015-2020.

9 https://www.agenda-2030.fr/actualites/feuille-de-route-de-la-france-pour-lagenda-2030-368.

10 Only Finland did not “translate” the internationally based SDG framework, instead they are using their sustainability strategy adopted in 2013 as well as the UN SDG indicators.
