# Supplementary material for: The politics of national SDG indicator systems: A comparison of four European countries
Source: Ambio. 2023 Feb 11;52(4):743–56. doi: 10.1007/s13280-022-01809-w (PMC9922038; doi:10.1007/s13280-022-01809-w)
Supplement: Supplementary file 1 — Supplementary file1 (PDF 838 kb) [file 13280_2022_1809_MOESM1_ESM.pdf]

Ambio
Electronic Supplementary Material
This supplementary material has not been peer reviewed.

Title: **The politics of national SDG indicator systems: a comparison of four European countries**

Authors: Robert Lepenies, Leonie Büttner, Ilona Bärlund, Kurt Jax
Jari Lyytimäki, Anders Branth Pedersen, Helle Ørsted Nielsen,
Claire Mosoni, Raoul Mille, Gerard Payen, Didier Richard

## ⇨ Appendix 1: Examples of appraisal mechanisms for national sustainability strategies in Germany and Finland

| | Institutions that are officially mandated to review the sustainable development strategy and policy | State/public institutions that proactively provide systematic reviews | Partly mandated appraisals & consultation processes | No mandate (non-governmental appraisals and shadow reports) |
|---|---|---|---|---|
| Germany | ⇨ Federal Government publishes progress reports on sustainable development and the implementation of the sustainability strategy (2004, 2008, 2012, 2014)<br>⇨ The Federal Ministries regularly issue departmental reports on the implementation of the National Sustainability Strategy.<br>⇨ Every two years, the Federal Statistical Office publishes an Indicator Report with an assessment of the achievement of the goals (2006, 2008, 2010, 2012, 2014, 2016, 2018, 2021) as well as an evaluation of the indicator progress every four years (2008)<br>⇨ The German Council for Sustainable Development has been commissioned by the Federal Government to organise an international peer review of German sustainability policy (2009, 2013, 2017)<br>⇨ Parliamentary Advisory Committee issues an opinion on the Sustainable Development Strategy (2017, 2019, 2020), the indicator reports (2007, 2010, 2014), progress reports (2004, 2008, 2012) and peer reviews (2010, 2013, 2018)<br>⇨ Voluntary National Report (VNR) 2016 by the Federal Government<br>⇨ Dialogue version of the national sustainability strategy (2020) | ⇨ The **Court of Auditors** has issued a report in 2018 on the objectives of sustainable development (Bonn Declaration on Sustainability)<br>⇨ **German Advisory Council on the Environment:** Statement on the Sustainability Strategy (2016)<br>⇨ **German Council for Sustainable Development:** Statement on the draft of the German Sustainability Strategy (2016) | ⇨ Consultation process with stakeholders organized by RNE for the Peers<br>⇨ **WPN2030** Consultation process<br>⇨ Statement from **RENN.** Regional network points Sustainability strategy. (2016) | ⇨ Statement on the German Sustainability Strategy by **BUND** (2016, 2018)<br>⇨ Statement of the **German Trade Union Confederation (DGB)** on the German Sustainability Strategy 2016.<br>⇨ **NABU** (2016), Opinions on the implementation of SDGs in Germany to the Federal Cabinet and all federal ministries<br>⇨ Statement of the German **Development Institute (DIE)** on the draft of the new German Sustainability Strategy (2016)<br>⇨ Statement of **MISEREOR** on the draft of the German Sustainability Strategy (2016)<br>⇨ Deutschland und die globale Nachhaltigkeitsagenda 2017. Großbaustelle Nachhaltigkeit. (Shadow Report) |

| | | | | |
|---|---|---|---|---|
| | ⇨ National Sustainability Strategy (2021) | | | |
| Finland | ⇨ National Commission on Sustainable Development responsible for coordination<br>⇨ Development Policy Committee responsible for issues related to foreign policy<br>⇨ Government National Implementation Plans for 2030 Agenda (given as a Report to the national Parliament), 2017 and 2020<br>⇨ Regular national monitoring and yearly reporting on the progress to the Parliament<br>⇨ Inter-ministry coordination netw ork<br>⇨ National netw ork for follow-up and review , including w ith the indicators (PMO)<br>⇨ VNR submitted to UN in 2016 and 2020<br>⇨ Statistics Finland coordinates and collects the data for SDG indicators<br>⇨ Prime Minister's Office<br>⇨ coordinates and collects the data for national indicators | ⇨ National Audit Office (2010, 2019 | ⇨ Citizen jury, a panel for sustainable development, composed of around 500 citizens, established in 2018 and renew ed in 2019 by a public call<br>⇨ Youth Group consisting of 20 young "sustainability ambassadors" | ⇨ Brief assessments by the Expert panel for Sustainable development<br>⇨ Examples of statements/ review s by NGOs? |

## ⇨ Appendix 2: Case study questionnaire

These are the questions that were asked in all four case studies (Denmark is taken here as an example)

DENMARK

Sustainability policy and indicator culture in Denmark

(1) Policy integration of SDG indicators on national level

- Policy integration of SDG indicators:
  - What SD indicator systems exist on the national level? What SD indicators existed before 2015?
  - How are the SDGs integrated into national policy plans and strategies; how aligned are they with the SDGs? To what extent are the comparable to global SDG indicators?
  - How many indicators are there?
- Development of the SDG indicator framework
  - When was the indicator framework first published? When was it updated? By whom?
  - Can we see a mainly data-driven approach or goal-driven approach? Who was involved in the development of the national indicator system? Was this an inclusive process / Citizen-based process / top-down process / bottom-up process / cooperation etc.?
- Important: What other aspects make this "indicator culture" special?

(2) Management of SDG indicators on the national level

- How is SDG/SD policy managed in broad terms (organizational chart)?
- How are SDG indicators managed on the national level? By whom and how?
- Who is responsible for reporting SDG indicators and national SD indicators?

Appraisal mechanism for sustainability strategy in Finland and the role of SDG indicators for environmental policy change

- What formal and informal appraisal mechanism exist to review the national sustainability strategy and the implementation of the SDGs? Do they exist? What is actually be done (year, link and actor)?
- What role/function is assigned to the (environmental) indicators? What is the political debate about the indicator and how visible are they in the SDG discussion?
- What role do indicators play in the implementation of the SDGs?
- What is the role of SDG indicators for (environmental) policy change in Denmark? Did the indicator lead to measures or new interventions in the field?

- How are the indicators used?

## ⇨ Appendix 3: Data sources used

| | Data sources |
|---|---|
| **National level indicators Finland** | - Indicator portal presenting the national SD indicators (Prime Minister's Office) and the national SDG indicators (Statistics Finland)<br>- **Selected national policy documents** (PMO 2017) and **assessment reports** of SD policies (Patosaari 2003; Ramboll 2009; Berg et al., 2019; VTV 2010; 2019)<br>- **Interviews**: Group interview of four senior officials of the Prime Minister's Office (Finland)<br>- **Participatory observations:** National Monitoring Network responsible for indicator development, coordinated by Prime Minister's Office (2017 onwards) |
| **National level indicators Germany** | - Indicator portal presenting the national SD indicators (Federal Statistical Office)<br>- **Selected national documents of SD policies:** National Sustainability Strategy (2002-2016); German Sustainable Development Strategy (since 2016); Indicator Reports of the Federal Statistical Office (since 2004); Dialogue version of National Sustainability Strategy (2020)<br>- **Selected assessment reports of SD policies:** German Council for Sustainable Development 2015, 2018a, 2018b, Parliamentary Advisory Committee on Sustainable Development 2017, 2018; German Advisory Council on the Environment 2016<br>- **Interviews**: Three interviews with stakeholders involved in the national SDG indicator process.<br>- Scientific articles on the national sustainability architecture, the German sustainable development strategy and the national SDG indicators |
| **National level indicators Denmark** | - **Indicator portal** presenting the national SD indicators (Statistics Denmark)<br>- **Selected national documents of SD policies:** Sustainable Development Strategy 2001, 2009, 2014)<br>- **Selected assessment reports of SD policies:** VNR 2018<br>- Interview with public employee in the Danish Ministry of Environment 2020 |

| | |
|---|---|
| National level indicators France | - **Indicator portal** presenting the national SD indicators (INSEE)<br>- **Selected national documents of SD policies:** SNTEDD 2015-2020, France´s new roadmap for the 2030 Agenda<br>- **Selected assessment reports of SD policies:**<br>- Interview with two members of Ministère de la Transition Ecologique et Solidaire-MTES; Interview with Gérard Payen (French Water Partnership), co-author of this paper |

Each country team was asked to report through a broad search strategy to what extent indicators formed part of political conversations about sustainable development (e.g. in the press, parliamentary proceedings or NGO communication). A drawback of such a broad empirical strategy is the difficulty for systematic comparison. Results from workshops and interviews with indicator professionals and potential users were employed to generate more in-depth understanding of perceptions of key actors of the role of indicators in national SDG policy. An expert validation was undertaken by scholars that belong to similar networks (e.g. the Partnership for European Environmental Research) and come from similar backgrounds (European Environmental Evaluators Network) which raises problems of endogeneity. In the Finnish case, additional insights were obtained through personal participation on the indicator preparation processes. In Germany and France, interviews were conducted with actors involved in the governance of SDG strategies, as well as the development process of the SDG indicator system. Questions were asked about the national SD indicator system, the actors involved in the development process and the challenges and potentials of the indicator system (while we could cover public actors systematically, it is possible that our coverage of civil society engagement with both SDG indicators and implementation processes is incomplete). The Danish case study is primarily based on a qualitative interview with a public employee from one of the Danish ministries central for the Danish SDGs and a comprehensive assessment of the Danish ministries' work with the SDGs implemented and published by the Danish state auditors – Rigsrevisionen (2020). After writing up the first draft, we revisited some interview partners for validation of our findings.

## ⇨ Appendix 4: National indicator systems – mapping overview

### o Denmark

In Denmark, the government is responsible for how to incorporate the SDGs into policies, and the Danish parliament – Folketinget – has not issued any legislation or guidelines related to the SDGs (Rigsrevisionen 2020). This means that the parliament does not formally oversee implementation of SDGs per se, but in so far as SDGs are implemented through other policies, Folketinget oversees the government's

implementation of the SDG, through its normal oversight mechanisms. The Danish government launched a first Sustainable Development Strategy in 2001 and the latest strategy in 2014 (Danish Environmental Protection Agency 2021). In 2017, the Liberal-Conservative Government approved a SDG action plan which announced the vision of "… maintaining a free, prosperous and safe Danish society and make a difference in the world around us" (Danish Government 2017a: 7). The plan translated all 17 SDGs into four priority areas: prosperity, people, planet, and peace and 37 national targets supported by 49 national indicators (1-2 indicators per target) were listed under these four priorities (Danish Government 2017a). The government selected indicators that were deemed relevant in a Danish context (European Parliament 2019). A model for assessing SDG consequences of proposals was developed by the Ministry of Finance and Ministry of Justice (Rigsrevisionen 2020). According to the European Parliament (2019) and the Danish Government (2017b) multiple actors (civil society, business, municipalities, youth, academia, primary and secondary schools) have been involved in the process on Danish SDGs. There were consultations and stakeholder conferences ahead of the introduction of the action plan, and a so called '2030 panel' was established with 22 members from different parts of society giving advice to members of parliament (EU 2019). In a common 2017 message from the Danish stakeholders they declared that they were invited to share expectations and wishes for the Danish SDGs, but also expected to be involved to a higher degree in future processes (Danish Government 2017b, p.41). After a change of government in mid-2019, there is no Danish SDG action plan, but a new plan was expected to be presented before the mid-2021 UN summit (Ministry of Finance 2020)

Historically, the Danish indicator system has been shaped through international collaboration on the SDGs in Nordic Council (EU 2019; Nordic Council 2003). The Ministry of Finance is responsible for coordinating the national implementation (see figure 01), while the Ministry of Foreign Affairs is responsible for implementing the SDGs in the UN context (and other international fora). The responsibility for follow-up on the individual 37 national targets has been divided among different ministries – a substantial part are with the Ministry of Food and Environment (Interview Danish Ministry of Environment and Food 2019; Rigsrevisionen 2020). Each line ministry has an international/EU department which links domestic and international monitoring. Since 2016, the SDGs have been integrated into the national annual bill as part of the budget for development cooperation (EU 2019). Danish subnational policies are separate from national policies (EU 2019) resulting in large variation in SDG attention in the municipalities. Responsibility for the more technical indicator follow-up on the SDG targets is located in Statistics Denmark (Rigsrevisionen 2020), where the work is divided among different offices and coordinated by a dedicated SDG unit with input from ministries. A parliamentary working group for the SDGs was placed under the auspices of the Finance Committee. The aim of this group is to ensure coordination between national and international aspects of the SDGs. Additionally, a non-partisan network, the 2030-network, which is open to all members of Parliament was established in 2017. Its main purpose is to facilitate information sharing and dialogue through meetings with government ministers and participation in public debates in Denmark and abroad, and has also initiated a baseline project. The network counts some high-level members of the Parliament.

- **Finland**

Finland is one of the pioneers of national level sustainable development policies. The Finnish National Commission on Sustainable Development (FNCSD) was established in 1993. The first Finnish Sustainable Development strategy was introduced in 1998 and the second in 2006. Both were rather extensive and detailed formal government documents while the current strategy is a concise charter approved by the FNCSD in 2013 and updated in 2016. The charter outlines vision for sustainability to 2050 and defines the general level guiding principles and eight goals. Starting from 2016, the responsibility for coordinating the national implementation of sustainable development policy and Agenda 2030 was shifted from the Ministry of the Environment to the Prime Minister's Office. This showed a recognition of sustainable development a holistic issue of well-being, not just an ecological issue. A new national roadmap for achieving the SDGs was expected to be approved by FNCSD in 2020 but the preparation was delayed mainly because of the management of COVID-19 pandemic.

The Finnish SDG indicators are compiled and published by Statistics Finland, the national agency responsible for compiling and verifying country data.[1] The set consists of global indicators as defined by the IAEG-SDGs and contains only the quantitative data (presented as graphical time-series) without qualitative interpretations. The first version, released in February 2019, included 405 individual time series. Data was missing from 32% of the Tier I indicators, 64% of the Tier II indicators and 79% of the Tier III indicators (VTV 2019). Most of the time series started from 1990 but the temporal coverage and timeliness of the data varies considerably. Another national set of sustainability indicators has been published by the Prime Minister's Office under the auspices of the National Commission on Sustainable Development.[2] This indicator set builds mainly on past national-level experiences on developing national level sustainable development indicators (Lyytimäki and Rosenström 2008; Rosenström 2018). It primarily aims to cover the eight goals outlined in the national sustainable development charter and only minor attention has been directed to compatibility with the 17 SDGs (Lyytimäki 2019b). The Finnish national goals have considerable overlaps with the 17 SDGs but the national indicators largely lack equivalents in the SDG indicators. This was a conscious choice aiming to better describe issues of national importance, and the outcome of historical policy debates about how sustainable development should be interpreted.

- **Germany**

Germany developed their first sustainability strategy quite late, in 2002. However, it aligned and completely overhauled its strategy with the SDGs. A new sustainability strategy, including sustainability indicators, was adopted by the German government in early 2017. It was developed in a joint coordination process in 2015/2016 with the participation of the Federal Statistical Office and the respective ministries and

---

[1] http://www.stat.fi/tup/kestavan-kehityksen-yk-indikaattorit-agenda2030_en.html

[2] https://kestavakehitys.fi/en/monitoring

an accompanying (limited) consultation process with stakeholders. Since then, the national sustainability strategy exists and can be regarded as the German translation of the SDGs. It has become the essential framework for the implementation of the 17 SDGs on the national level (Blumers and Kaumanns 2017).

Both the responsibility for reporting the global SDG indicators and the national SD indicators lies both with the Federal Statistical Office, even though each line ministry is responsible for providing data and for implementing goals that were specifically assigned to them. The Federal Statistical Office coordinates the data on the global SDG indicators and publishes the data every year.[3] In addition, an indicator report with the evaluation of the national SD progress is published every two years. The Federal Statistical Office is in charge of examining the updated list of national indicators introduced in 2018 (Federal Government 2018).
While the responsibility for reporting lies with the Federal Statistical Office, regarding the management of SDG indicators, a highly sectoral way of thinking can be observed. Based on our interviewees, opportunities to work together across ministries – on partly interlinked SDGs – are rarely used (Interview BMUB). This is especially so in the case of environmental challenges and their respective indicators. External stakeholders view this similarly, but add that the larger problem is the lack of executive political party decisions to endow the sustainability strategy with sufficient energy (Interview BMUB). Beside the lack of commitment, administrative structures as well as resources are often insufficient to implement the planned measures, for example, measures in the course of the WFD (Interview BMUB). It is however noteworthy that the 2021 strategy includes a new emphasis on the need for sustainability transformations, most due to input from experts at the science-policy interface (e.g. SDSN, German Institute for International and Security Affairs (SWP) and others). The 2021 strategy now closely corresponds to conceptualizations within UN Global Sustainable Development Report (GSDR 2019). Equally important, it now includes designated "Transformationsbereiche" (transformation areas) that include wellbeing/capabilities; energy/climate; circular economy; construction/traffic; food/agriculture; zero pollution) as well as new language on key transformation indicators ("Schlüsselindikatoren") and increased focus on off-tracks indicators. It also includes new indicators (some Covid-related), as well as new indicators that the coalition government wants to highlight which align with their agreed upon political agenda. Our interviews suggest that it is mostly due to input from the scientific community in Germany that the language of transformation is now firmly embedded in the strategy in 2021.

Prior to Agenda 2030, the national sustainability strategy (“DNS”, Deutsche Nachhaltigkeitsstrategie) was divided into four areas (intergenerational justice, quality of life, social cohesion and international responsibility) and contained 38 sustainability indicators which were mostly associated with quantified goals. With the rewriting of the sustainability strategy, not only was the division into sustainability areas re-aligned to the SDGs, but new themes were also included (including poverty, water, sanitation, consumption and production patterns and

[3] www.destatis.de/SDGDE

oceans). Despite this overhaul, most of the indicators that had been used previously were also unaltered in the new strategy. It was rather that new indicators were added to the strategy (not all of them were developed and set up without conflict – indeed, in political and value negotiations are occurring in the shadow of these indicators). Since 2018 the sustainability strategy contains 66 indicators (Federal Government 2021), of which about half are similar to the global SDG indicators (Blumers and Kaumanns 2017). The reduced number was selected based on a data-driven approach (leading to mostly quantitative indicators) and in a top-down process which was criticized for its lack of transparency. We find that the selection of indicators for the sustainability strategy was largely left to statisticians, who agreed on them in consultation with the relevant ministries. As the 2021 dialogue version on the further development of the German Sustainability Strategy once again states, statisticians in Germany should take over the analysis of indicator development "independently and under their own professional responsibility" (Federal Government 2021). A new and expanded version of the strategy was announced in March 2021 that substantially responded to critique from scientific commentators and that fortified language on "sustainability transformations" that were necessary to attain in German sustainability policy. (Federal Government 2021b).

- **France**

In France, the national preexisting sustainability strategy, revised some months before the establishment of the Agenda 2030 and the SDG framework in 2015, was called the national strategy for an ecological transition to sustainable development 2015-2020 - La stratégie nationale de transition écologique vers un développement durable (SNTEDD)[4]. It was adopted by the Council of Ministers in February, 2015, six months before the adoption of the UN resolution on Agenda 2030 and the SDG framework by the United Nations (September 2015). Deliberations about a new national SDGs strategy started separately in 2017 (Aubert et al. 2017) – as this new strategy included the objectives of SNTEDD (which was mainly ecologically-oriented, and provided a roadmap until only 2020) as well as other (socio-economic) sustainability objectives, the SNTEDD was replaced *de facto*. This new document was released in September 2019 and represents France´s new roadmap[5] for the 2030 Agenda.

Table 2: The ten wealth indicators and their inclusion in French, UN and EU goals and indicators

[4] https://www.ecologique-solidaire.gouv.fr/strategie-nationale-transition-ecologique-vers-developpement-durable-2015-2020
[5] https://www.agenda-2030.fr/actualites/feuille-de-route-de-la-france-pour-lagenda-2030-368

| Sector | Themes | Present well-being | Future well-being | "Elsewhere" well-being | France SDG | UN SDG | UN-EU |
|---|---|---|---|---|---|---|---|
| **Economic** | Job | 1. Employment rate of 15-64 year olds | | | 8.i.2 | Goal 8 | SDG_08_30 |
| | Investment | | 2. GERD / GDP research expenditure | | 9.i.5 | 9.5.1 | SDG_09_10 |
| | Financial stability | | 3. Public and private debt | | 17.i.3 | 10.5.1 | SDG_17_40 |
| **Social** | Health | 4. Healthy life expectancy | | | 3.i.1 | Goal 3 | SDG_03_10 |
| | Life satisfaction | 5. Overall satisfaction with life | | | 3.i.4 | no | no |
| | Inequality | 6. Income dispersion | | | 10.i.2 | Goal 10 | SDG_10_41 |
| | Poverty | 7. Poverty rate in living conditions | | | 1.i.2 | 1.2.1 | SDG_01_30 |
| | Education | 8. Early school leavers | | | 4.i.4 | 8.6.1 | SDG_08_20 |
| **Environmental** | Climate | | | 9. Carbon footprint | 13.i.3 | Goal 13 | no |
| | Biodiversity | | 10. Soil artificialization | | 11.i.2 | Goal 15/11 | no |

Table based on https://www.insee.fr/fr/statistiques/3281635?sommaire=3281778#consulter – this exemplifies a national translation of the SDGs for the French context.

After the release of the global SDG indicators in 2017, values for 120 SDG indicators were first published corresponding to the global SDG indicators available in France. However, those first reported values were based on previous data availability and were not used for national assessment of sustainable development (Interview MTES). To have an effective set of national SDG indicators, the National Council for Statistical Information (CNIS), in collaboration with INSEE and SDES, proposed a mandate in June 2017 for a working group to establish a set of SDG indicators that would track progress on the main SDG targets that are of relevance in France. Made up of more than 100 participants, this working group gathered people from a wide range of backgrounds (civil society, stakeholders, ministerial and statistical services). Following this work, a dashboard of 98 SDG indicators was proposed in mid-2018 which would constitute the national framework for monitoring France's progress in achieving the 17 SDGs (CNIS 2018). The French dashboard thus contains a number of UN global indicators deemed to be the most suitable for the national context, as well as the 10 new wealth indicators (also considered as SDG indicators and thus, published by INSEE like all the other SDG indicators) and additional indicators specific to French particularities, among which are some SNTEDD monitoring indicators

(Vey and Hesse 2019). The 98 national SDG indicators are available publicly, and commented upon, on the INSEE website.[6] They are reported and managed by the ministry relevant to the indicator before being shared with INSEE for publication.

[6] https://www.insee.fr/fr/statistiques/2654964
